# Adaptation to altitude affects the senescence response to chilling in the perennial plant *Arabis alpina*

**DOI:** 10.1093/jxb/eru426

**Published:** 2014-11-04

**Authors:** Astrid Wingler, Marta Juvany, Caroline Cuthbert, Sergi Munné-Bosch

**Affiliations:** ^1^Genetics, Evolution and Environment, University College London, Gower Street, London WC1E 6BT, United Kingdom; ^2^Departament de Biologia Vegetal, Facultat de Biologia, Universitat de Barcelona, Avinguda Diagonal 643, E-08028 Barcelona, Spain

**Keywords:** *Arabis alpina*, cold stress, jasmonic acid, phytohormones, senescence, sucrose, zeatin.

## Abstract

In annual plants with determinate growth, sugar accumulation signals high carbon availability once growth has ceased, resulting in senescence-dependent nutrient recycling to the seeds. However, this senescence-inducing effect of sugars is abolished at cold temperature, where sugar accumulation is important for protection. Here, natural variation was exploited to analyse the effect of chilling on interactions between leaf senescence, sugars, and phytohormones in *Arabis alpina*, a perennial plant with indeterminate growth. Eight accessions of *A. alpina* originating from between 2090 and 3090 m above sea level in the French Alps were used to identify heritable adaptations in senescence, stress response, sugars, and phytohormones to altitude. Accessions from high altitudes showed an enhanced capacity for sucrose accumulation and a diminished loss of chlorophyll in response to chilling. At warm temperature, sucrose content was negatively correlated with chlorophyll content, and sucrose treatment induced leaf senescence. Chilling resulted in lower indole-3-acetic acid, but higher zeatin and jasmonic acid contents. Interactions between sugar and phytohormones included a positive correlation between sucrose and jasmonic acid contents that may be involved in promoting the stress-dependent decline in chlorophyll. These findings reveal regulatory interactions that underlie adaptation in the senescence and stress response to chilling.

## Summary

Plants originating from high altitude have an increased capacity for sucrose accumulation in response to chilling and sucrose content is positively correlated with jasmonic acid in accessions from different altitudes.

## Introduction

During leaf senescence, nutrients (e.g. nitrogen) are recycled from the old leaves. In monocarpic annual plants, which only flower once, the main function of leaf senescence is the provision of nutrients for seed formation before death of the whole plant (Davies and Gan, 2012). Perennial polycarpic plants survive the reproductive phase, but individual leaves senesce nevertheless: in deciduous perennials senescence occurs seasonally, often in autumn, and nutrients are stored over winter; in evergreen perennials some green leaves are maintained, and only the old leaves senesce, but leaf longevity can vary widely between species. *Arabis alpina* is a polycarpic, evergreen perennial with short-lived leaves (Wingler *et al.*, 2012*a*). It is related to *Arabidopsis thaliana* (*Arabidopsis*) and has been established as a model for flowering in perennial plants (e.g. Bergonzi *et al.*, 2013). Similar to *Arabidopsis*, flowering branches of *A. alpina* die soon after seed formation, but vegetative branches continue to grow, requiring senescence-dependent nutrient recycling from the old to the young leaves.

The carbon dynamics during senescence of *Arabidopsis* and *A. alpina* plants are likely to be different. In *Arabidopsis*, rosette growth ceases during the reproductive phase, whereas photosynthesis initially continues. In addition to the leaf rosette, the photosynthetic inflorescence supplies carbon, providing over half of the lifetime carbon gain of the plant (Earley *et al.*, 2009). Carbon is therefore not limiting and accumulates in the rosette leaves in the form of sugars (e.g. Wingler *et al.*, 2012*b*). This sugar accumulation can promote senescence in *Arabidopsis* and other annual plants with determinate growth. Cultivation of *Arabidopsis* plants in the presence of glucose in combination with low nitrogen supply does not only induce leaf yellowing, but also results in gene expression patterns that are characteristic of developmental leaf senescence (Pourtau *et al.*, 2006; Wingler and Roitsch, 2008; Wingler *et al.*, 2009). Owing to the different carbon dynamics, the role of sugars in regulating senescence in perennial plants with indeterminate growth is less clear than in *Arabidopsis*. In *A. alpina*, sugars only accumulated with leaf age when the plants were grown under controlled conditions at warm temperature, whereas sugars declined with leaf age in the field. In addition, the senescence response to glucose treatment was less pronounced than in *Arabidopsis*, and varied between *A. alpina* accessions (Wingler *et al.*, 2012*a*).

Leaf sugar contents typically increase during chilling (e.g. Cook *et al.*, 2004), where sugars have an osmoprotective function. Further, sucrose has been shown to play a role in the cold acclimation pathway (Rekarte-Cowie *et al.*, 2008) and glucose treatment up-regulates expression of cold-regulated (*COR*) genes (Masclaux-Daubresse *et al.*, 2007). It is important that this accumulation of sugars does not result in the induction of premature senescence. Although severe stress usually results in stress-related senescence, moderate cold stress can delay leaf senescence in *Arabidopsis* and cultivation of *Arabidopsis* plants at cold temperature inhibits sugar-induced leaf senescence (Masclaux-Daubresse *et al.*, 2007). Overexpression of the C-repeat binding factor CBF2, a transcription factor involved in cold acclimation, has also demonstrated a function of cold acclimation in the delay of leaf senescence. Plants overexpressing CBF2 showed delayed developmental senescence, as well as delayed induction of senescence by treatment with the phytohormones ABA, ethylene, salicylic acid, and methyl jasmonate (Sharabi-Schwager *et al.*, 2010). Expression of cold-regulated (*COR*) genes was identified as a possible downstream factor involved in the delay of senescence (Wingler, 2011). This was confirmed by overexpression of the *COR* genes *COR15A* and *COR15B* in *Arabidopsis*, which resulted in delayed senescence (Yang *et al.*, 2011). Surprisingly, the senescence-delaying effect of cold temperature is not as pronounced in *A. alpina* as in *Arabidopsis* (Wingler *et al.*, 2012*a*), despite the fact that *A. alpina* should be adapted to low temperatures occurring at high altitudes.

Phytohormones are involved in the regulation of leaf senescence and in the response to various biotic and abiotic stresses, including cold stress (Jibran *et al.*, 2013; O’Brien and Benková, 2013). Whereas ethylene, abscisic acid (ABA), jasmonates, and salicylic acid (SA) promote senescence, cytokinins, auxins, and gibberellins normally retard it (Lim *et al.*, 2007; Jibran *et al.*, 2013). There is crosstalk between sugar and phytohormone signalling pathways, including interactions with ABA, ethylene, cytokinin, and auxin (Moore *et al.*, 2003; Gibson, 2004). This may have consequences for senescence regulation. For example, a substantial overlap between glucose- and ABA-regulated gene expression was found in *Arabidopsis* (Li *et al.*, 2006). This includes the induction of senescence-associated genes, even though gene expression was analysed in seedlings. Work with ABA synthesis and ABA signalling mutants suggests that interactions between ABA and glucose are more important in seedlings than in mature plants (Pourtau *et al.*, 2004).

Changes in phytohormone contents during leaf senescence in perennial plants are generally in agreement with their function in annual plants. For example, in the perennial herb *Borderea pyrenaica*, ABA content increased during leaf senescence, whereas the contents of cytokinins and auxin decreased (Oñate *et al.*, 2012). In mastic trees (*Pistacia lentiscus*) the content of the cytokinin zeatin was reduced in older compared with younger plants and negatively correlated with the chlorophyll *a*/*b* ratio (Juvany *et al.*, 2013). However, little is known about how temperature and phytohormones interact in regulating leaf senescence in perennial plants or about the interactions with sugar signalling.

Natural intra-specific variation can be used to determine the relationship between traits in plants. A lot of recent work has focused on *Arabidopsis*, e.g. analysing the relationship between metabolism and stress response (Hannah *et al.*, 2006) or between different life-history traits such as senescence and flowering (Levey and Wingler, 2005). Differences in metabolite contents, including sugars, were found between populations of *Arabidopsis lyrata* ssp. *petraea* from northern Europe (Davey *et al*., 2008, 2009). Some studies have exploited natural variation to determine adaptations to the growth habitat along geographical gradients, including variation of flowering and growth phenotypes along altitudinal gradients in *Arabidopsis* (Méndez-Vigo *et al.*, 2011; Montesinos-Navarro *et al*., 2011). For alpine plants, the literature is, however, contradictory as to whether or not there is genetic adaptation along altitudinal gradients. Although Frei *et al.* (2014) found no adaptation to altitude of origin and concluded that phenotypic plasticity allows plants to grow at a range of altitudes, Gonzalo-Turpin and Hazard (2009) identified local adaptations despite phenotypic plasticity and gene flow between populations.

Temperature is a major driver of genetic variation in *A. alpina* (Manel *et al.*, 2010; Poncet *et al.*, 2010), but functional studies are still required to identify the physiological processes that determine temperature tolerance. Here, natural variation in *A. alpina* was exploited to determine the relationship between temperature, senescence, sugar, and phytohormone contents. Physiological adaptations to the altitude of origin were found, such as accumulation of more sucrose at cold temperature in plants from higher altitudes. Interactions between sugar accumulation and leaf senescence were identified, in addition to correlations between sugars and phytohormones.

## Materials and methods

### Plant material

*Arabis alpina* (L.) seed was collected from different populations in the Écrins region in the French Alps (Table 1). The maximum distance between the sites was 12.2 km. Minimum daily temperatures during the growing season decreased with a gradient of ca. 0.6–0.7 °C per 100 m altitude change (Supplementary Fig. S1). Seeds for individual accessions were produced by two rounds of selfing in the laboratory. Seeds were stratified for 5 d at 4 °C before sowing onto Levington cactus compost (Scotts-Miracle Gro, UK) mixed at a 2:1 ratio with perlite. All plants were initially grown at 20 °C with 12h of illumination per day at 100 µmol m^–2^ s^–1^. After 63 d, half of the plants were transferred to 5 °C with 12h of illumination per day at 80 µmol m^–2^ s^–1^. Two accessions (Lautaret-1 and Romanche) were discarded because of poor growth.

**Table 1.** Origin of the *A. alpina* accessions used

Site		
Romanche	GPS coordinates	Altitude (m.a.s.l.)
Lautaret-2	N 45°01’56.6’’, E 006°21’22.1’’	1684
Lautaret-1	N 45°01’57.6’’, E 006°24’20.9’’	2090
Ruillans-1	N 45°01’38.6’’, E 006°23’12.9’’	2093
Galibier-3	N 45°01’38.8’’, E 006°17’02.7’’	2359
Ruillans-6	N 45°03’08.1’’, E 006°23’14.8’’	2376
Galibier-1	N 45°01’39.9’’, E 006°16’55.9’’	2379
Galibier-4	N 45°03’39.9’’, E 006°24’16.8’’	2556
Pic Blanc	N 45°03’38.9’’, E 006°24’03.2’’	2655
Ruillans-2	N 45°03’50.3’’, E 006°23’06.0’’	2911

For growth on agar plates, seeds (accessions Galibier-4, Lautaret-1, Lautaret-2, Pic Blanc, Romanche and Ruillans-2) were sown onto agar (1% w/v) plates (25ml medium per 9cm diameter Petri dish) with low nitrogen (4.7mM NO_3_^–^) medium (Wingler *et al.*, 2004) with or without addition of 55.5mM sucrose. After stratification for 2 d of at 4 °C, the plates were arranged vertically in growth chambers at 20 °C with 12h of illumination per day at 150 µmol m^–2^ s^–1^.

### Determination of chlorophyll content and maximum photosystem II efficiency (F_v_/F_m_)

Leaves were counted from the base and marked with coloured thread. For all plants, measurements were initially performed on leaf 12 and, if large enough, leaf 14. Once leaf 12 had died, measurements were continued with leaves 14 and 16. Chlorophyll content was determined with a CCM-200 chlorophyll content meter (Opti-Sciences, Hudson, USA) and F_v_/F_m_ with a FMS-2 pulse-modulated fluorometer (Hansatech, King’s Lynn, UK). For plants grown on agar plates, whole-rosette F_v_/F_m_ was imaged using a FluorCam 700MF kinetic imaging fluorometer (Photon Systems Instruments, Brno, Czech Republic) as described by Wingler *et al.* (2004).

### Determination of sugar and hormone contents

Two leaves were harvested for each plant on day 92, one day after determination of chlorophyll content and F_v_/F_m_. Two leaf positions were chosen for each accession to capture different leaf developmental stages, a fully expanded leaf without visible senescence (mature) and a leaf two ranks lower along the stem (old). The exact position chosen was dependent on the developmental stage of each accession at a given temperature: For all cold-treated plants, leaf 14 was harvested as “mature” leaf and leaf 12 as “old” leaf. For warm-treated plants, leaves 14 (mature) and 12 (old) were harvested for the accessions Lautaret-2, Ruillans-1, and Ruillans-6; leaves 16 (mature) and 14 (old) were harvested for the accessions Galibier-1, Galibier-3, Galibier-4, Pic Blanc, and Ruillans-2. Leaves were frozen in liquid nitrogen and stored at –80 °C until processing for phytohormone and sugar analyses. Values in these samples are expressed on a leaf fresh weight basis. Additional leaves of the same plants were identified based on their chlorophyll content on day 93 and divided into “green” and “senescent” leaves. Discs were cut from these leaves on day 94 for determination of sugar content expressed on a leaf area basis. All material was harvested around 6h into the photoperiod.

The extraction and analyses of endogenous contents of the cytokinins, zeatin (Z), zeatin riboside (ZR), isopentenyladenosine (iPA), 2-isopentenyladenine (2iP), as well as indole-3-acetic acid (IAA), gibberellins (GAs), abscisic acid (ABA), salicylic acid (SA), jasmonic acid (JA), and the ethylene precursor 1-aminocyclopropane-1-carboxylic acid (ACC) were carried out using ultrahigh-performance liquid chromatography coupled to electrospray ionization tandem spectrometry (UHPLC/ESI-MS/MS) as described by Müller and Munné-Bosch (2011). Deuterium labelled phytohormones were used as internal standards. Sugars were extracted at 80 °C in 80% ethanol and determined spectrophotometrically using coupled enzymatic assays (Stitt *et al.*, 1989) in a microplate reader.

### Statistics

Correlations and regressions were analysed using Minitab 15. For correlation between sugars, hormones and chlorophyll each plant (biological replicate) was treated separately, and correlations are based on measurements of the different parameters in the same leaves. For regression between altitude and measured parameters, replicate plants for each treatment and developmental state were averaged. Differences between accessions were analysed using one-way ANOVA, followed by Tukey’s pairwise comparison using SPSS 14.0.

## Results

### Effect of chilling on stress response and senescence

**Fig. 1.**
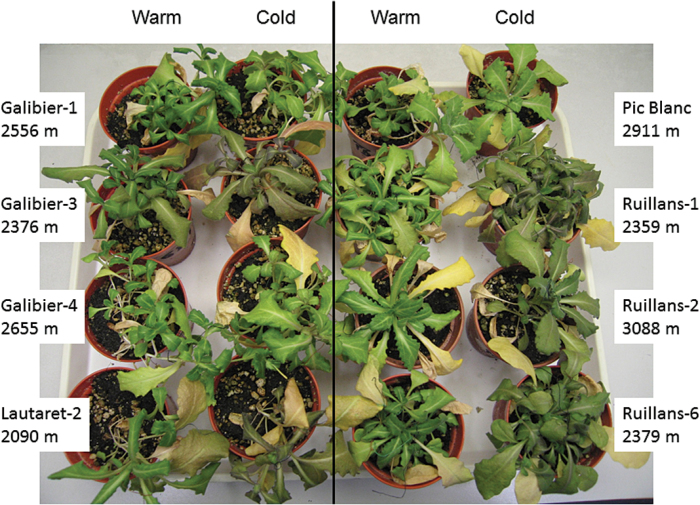
*A. alpina* plants on day 88 after continuous growth at 20 °C (warm) or after transfer to 5 °C on day 63 (cold).

**Fig. 2.**
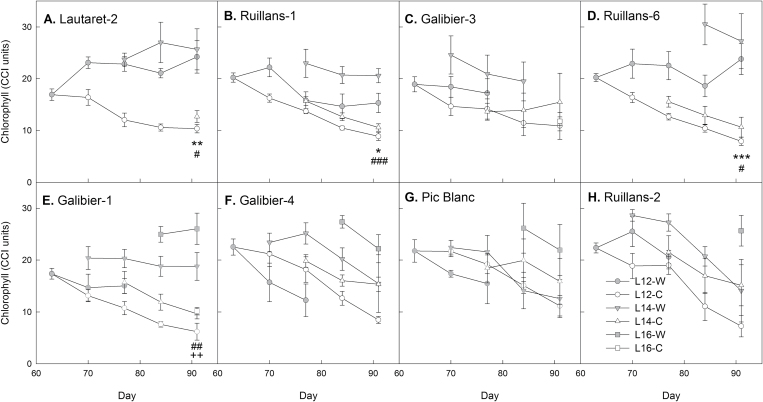
Chlorophyll content in the leaves of *A. alpina* plants grown continuously at 20 °C (warm) or transferred to 5 °C on day 63 (cold). Locations are presented in order of increasing altitude of origin (A–H) L12-W=leaf 12, warm (grey circles); L12-C=leaf 12, cold (white circles); L14-W=leaf 14, warm (grey triangles); L14-C=leaf 14 cold (white triangles); L16-W=leaf 16, warm (grey squares); L16-C=leaf 16 cold (white squares). Data are means of leaves from three to five plants±SEM. T-tests were performed to compare data from warm- and cold-treated plants at the final time point; asterisks indicate statistically significant differences between leaves 12 (* *P*≤0.05; ** *P*≤0.01; *** *P*≤0.001); hash signs indicate statistically significant differences between leaves 14 (^#^ *P*≤0.05; ^##^ *P*≤0.01; ^###^ *P*≤0.001); plus signs indicate statistically significant differences between leaves 16 (^+^ *P*≤0.05; ^++^ *P*≤ 0.01; ^+++^ *P*≤ 0.001).

All plants were initially grown for 63 d at warm (20 °C) temperature before chilling treatment (5 °C). Flowering in *A. alpina* is normally dependent on vernalization, and plants therefore remained in the vegetative state, although some warm-grown individuals of the Galibier-4 accession flowered without vernalization. Chilling induced anthocyanin accumulation in some of the accessions, especially in those from lower altitudes: Lautaret-2, Ruillans-1, and Galibier-3 (Fig. 1). Chlorophyll (Fig. 2) and F_v_/F_m_ (Supplementary Fig. S2) were monitored to determine the initial stress response and the subsequent senescence-dependent decline in photosynthetic function. For all plants leaf 12 (from the base) was initially used, but because of death of this leaf in some accessions, the younger leaves 14 and 16 were included if available. When leaves of the same position within each accession were compared, chlorophyll declined more rapidly at cold than at warm temperature in some of the accessions (Galibier-1, Lautaret-2, Ruillans-1, and Ruillans-6), resulting in significantly lower chlorophyll content at the final time point (Fig. 2). This response was, however, not observed in all accessions, most notably not in those from the highest altitudes, Pic Blanc and Ruillans-2. To analyse the relationship between altitude and the effect of temperature on chlorophyll content, regression analysis was conducted for time points when the same leaf position was available for all accessions (Table 2). Opposite relationships were found for warm and cold temperature. At warm temperature, regressions between chlorophyll content and altitude of origin were negative, and this relationship became statistically significant at the final time point (day 91), indicating that senescence in accessions from higher altitude had progressed further. In contrast, positive relationships were found at cold temperature, and these were significant for day 77. On days 70 and 77, chlorophyll content at cold temperature was highest in the three accessions from the highest altitudes, Galibier-4, Pic Blanc and Ruillans-2 (Supplementary Fig. S3), also indicating that accessions from higher altitude showed a less severe stress response to cold temperature.

**Table 2.** Relationship between chlorophyll content and altitude of originOnly time points for which leaves of the same position were available for all accessions are included. For regression analysis replicate plants of each accession were averaged. Values are coefficients of determination (*R* ^*2*^) with *P* values given in parentheses; statistically significant correlations are indicated in bold.

A. Warm		B. Cold	
	Altitude		Altitude
Chlorophyll Leaf 12, 70 d	negative 0.003 (0.892)	Chlorophyll Leaf 12, 70 d	positive 0.347 (0.124)
Chlorophyll Leaf 12, 77 d	negative 0.085 (0.482)	Chlorophyll Leaf 12, 77 d	**positive 0.648 (0.016)**
Chlorophyll Leaf 14, 91 d	**negative 0.617 (0.021)**	Chlorophyll Leaf 14, 91 d	positive 0.257 (0.200)

### Effect of chilling and leaf age on sugar accumulation

**Fig. 3.**
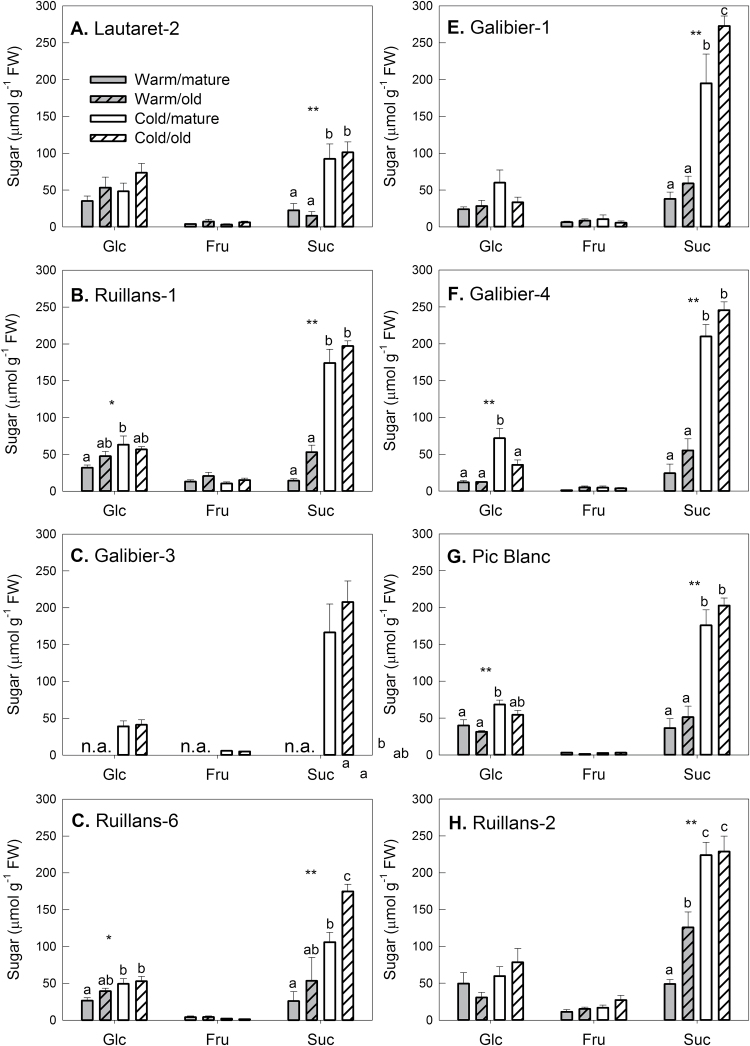
Glucose, fructose, and sucrose contents on day 92 in the leaves of *A. alpina* plants grown continuously at 20 °C (warm, grey) or transferred to 5 °C on day 63 (cold, white). Locations are presented in order of increasing altitude of origin (A–H) Dependent on the accession (see Materials and methods) leaf 14 or leaf 16 was classified as mature (open bars) and leaf 12 or leaf 14 as old (hashed bars). Data are means of leaves from three to five plants+SEM. Asterisks indicate statistically significant differences between the temperature treatments or age groups (ANOVA; *P≤ 0.05; **P≤ 0.01); different letters indicate differences between the groups (Tukey’s test). n.a.=not available.

Our previous work had shown that senescence of *A. alpina* leaves at warm temperature is accompanied by sugar accumulation, whereas sugar contents declined with leaf age at cold temperature or in the field (Wingler *et al.*, 2012*a*). As natural variation was found in this response, the sugar response to temperature was explored in a larger range of accessions, also comparing leaves of two different developmental stages, leaves 14 or 16 (mature) and leaves 12 or 14 (old), dependent on development of each of the accessions (Fig. 3). Results for Galibier-3 at warm temperature are not included as only two healthy plants were available. Chlorophyll content and F_v_/F_m_ were determined the day before the harvest (see day 91 in Fig. 2 and Supplementary Fig. S2).

Fructose contents remained low in all accessions, but some accessions showed accumulation of glucose in response to chilling (Fig. 3). No significant effect of leaf age on glucose content was found. Only Ruillans-2 contained significantly more sucrose in the old than in the mature leaves at warm temperature. Sucrose content generally increased in response to chilling. This was also found in a separate set of samples harvested 2 d later (Supplementary Fig. S4). For this harvest, leaves were not chosen according to position, but selected based on chlorophyll content (Supplementary Fig. S5) to allow comparison of green and senescent leaves. In these samples, sucrose content at warm temperature was significantly increased in senescent compared with green leaves of Ruillans-1 and Ruillans-6.

**Fig. 4.**
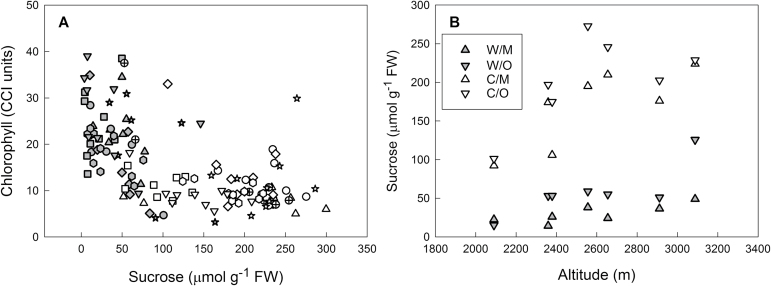
Relationship between sucrose content on day 92 and chlorophyll (A) or altitude of origin (B) in the leaves of *A. alpina* plants grown continuously at 20 °C (warm, grey) or transferred to 5°C on day 63 (cold, white). (A) Symbols represent individual plants; different accessions are represented by different symbols. Overall correlation: *r*=–0.615, *P*≤0.001; warm: *r*=–0.461, *P*≤0.001; cold: *r*=–0.282, P=0.016. (B) Replicate plants for each accession and leaf developmental stage were averaged for regression analysis. Regression warm: *R* ^*2*^=0.380, *P*=0.019; cold: *R* ^*2*^=0.404, *P*=0.008.W/M=warm/mature; W/O=warm/old.; C/M=cold/mature; C/O=cold/old.

To explore the relationship between sucrose content and senescence further, correlation analyses were conducted (Fig. 4A). Statistically significant negative correlations between sucrose and chlorophyll contents were found when all samples were considered and also for warm-grown plants treated separately, but correlation was less significant for cold-grown plants. This relationship was confirmed in an independent set of samples (Supplementary Fig. S6A), indicating that the senescence-dependent decline in chlorophyll at warm temperature is associated with sucrose accumulation.

Because for both sets of samples accessions from higher altitude showed stronger sucrose accumulation in response to chilling (Fig. 3 and Supplementary Fig. S4), the relationship between altitude of origin and sucrose was also analysed. Regression analysis showed a statistically significant positive relationship between sucrose content after chilling and altitude in both sets of samples (Fig. 4B and Supplementary Fig. S6B). At warm temperature, the regression was, however, only significant for the samples shown in Fig. 4B and not for the ones shown in Supplementary Fig. S6B. The gradient of the linear regression line at cold temperature was twice that at warm temperature, confirming that plants originating from higher altitudes have a higher capacity to accumulate sucrose during chilling. No statistically significant regressions between glucose or fructose and altitude were found.

### Interactive effects of sucrose and chilling on senescence

**Fig. 5.**
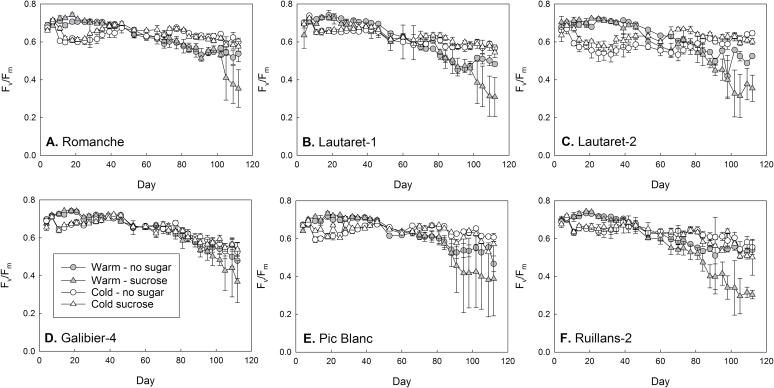
Effect of sucrose on maximum photosystem II efficiency (F_v_/F_m_) in whole rosettes of *A. alpina* plants grown continuously at 20 °C (warm, grey) or transferred to 5 °C on day 7 (cold, white). Plants were grown on low-nitrogen agar medium without or with addition of 55.5mM sucrose. Data are means of three to five plates±SEM.

Glucose treatment was shown to induce senescence in some but not all accessions of *A. alpina*, and no interaction between glucose and temperature was identified (Wingler *et al.*, 2012*a*). Based on our findings that sucrose accumulates strongly in response to chilling (Fig. 3 and Supplementary Fig. S4) and at warm temperature is correlated with the senescence-dependent decline in chlorophyll (Fig. 4A, Supplementary Fig. S6A), the interactive effects of sucrose and temperature was determined (Fig. 5). For this, plants were grown on agar plates with and without 55.5mM sucrose and whole-rosette F_v_/F_m_ was imaged. After an initial decline in response to transfer to 5 °C, F_v_/F_m_ recovered. Values then declined at warm temperature, in particular in the presence of sucrose, indicating senescence. However, senescence was not induced by sucrose at cold temperature. These findings show that the chilling-related sucrose accumulation is unlikely to trigger senescence, whereas an age-related increase in sucrose could accelerate senescence at warm temperature.

### Effect of chilling and leaf age on phytohormone contents

**Fig. 6.**
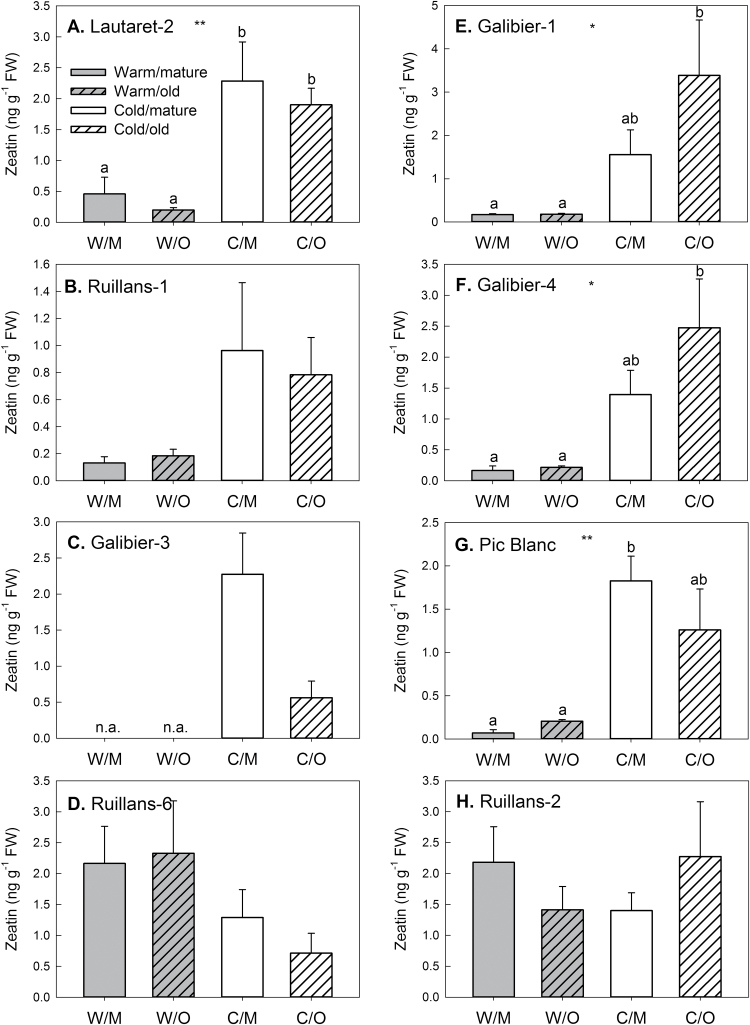
Zeatin content on day 92 in the leaves of *A. alpina* plants grown continuously at 20 °C (warm, grey) or transferred to 5 °C on day 63 (cold, white). Locations are presented in order of increasing altitude of origin (A–H) Dependent on the accession (see Materials and methods) leaf 14 or leaf 16 was classified as mature (open bars) and leaf 12 or leaf 14 as old (hashed bars). W/M=warm/mature; W/O=warm/old.; C/M=cold/mature; C/O=cold/old. Data are means of leaves from three to five plants+SEM. Asterisks indicate statistically significant differences between the temperature treatments or age groups (ANOVA; *P≤0.05; **P≤0.01); different letters indicate differences between the groups (Tukey’s test). n.a.=not available.

**Fig. 7.**
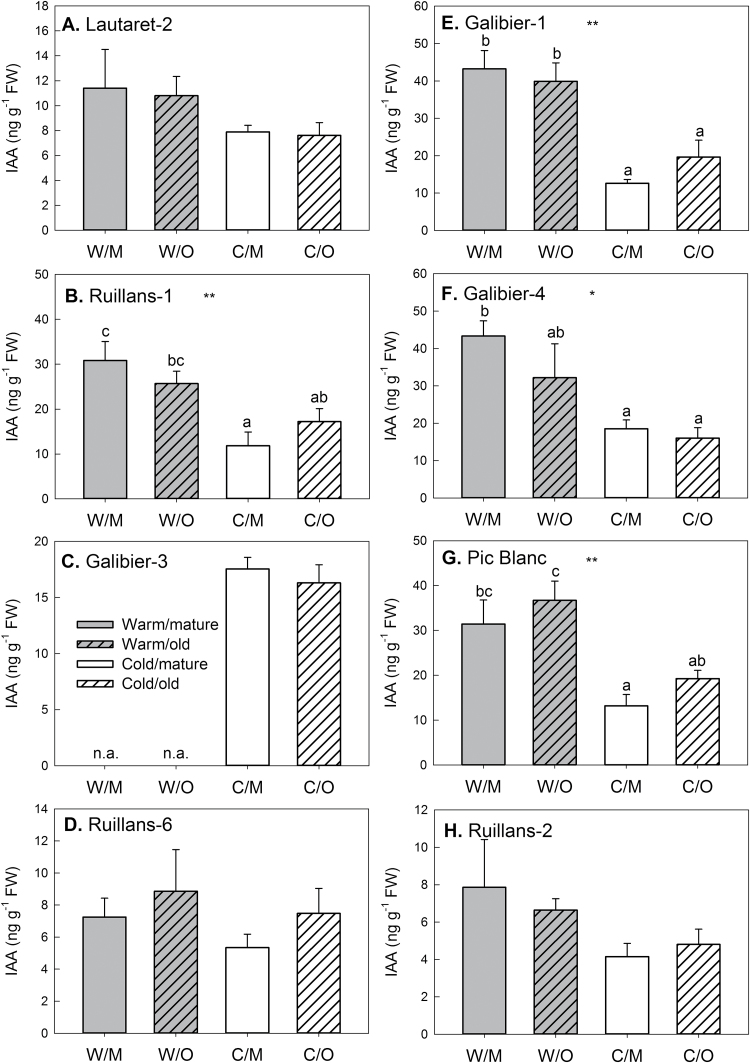
Indole-3-acetic acid (IAA) content on day 92 in the leaves of *A. alpina* plants grown continuously at 20 °C (warm, grey) or transferred to 5 °C on day 63 (cold, white). Locations are presented in order of increasing altitude of origin (A–H) Dependent on the accession (see Materials and methods) leaf 14 or leaf 16 was classified as mature (open bars) and leaf 12 or leaf 14 as old (hashed bars). W/M=warm/mature; W/O=warm/old.; C/M=cold/mature; C/O=cold/old. Data are means of leaves from three to five plants+SEM. Asterisks indicate statistically significant differences between the temperature treatments or age groups (ANOVA; *P≤ 0.05; **P≤ 0.01); different letters indicate differences between the groups (Tukey’s test). n.a.=not available.

**Fig. 8.**
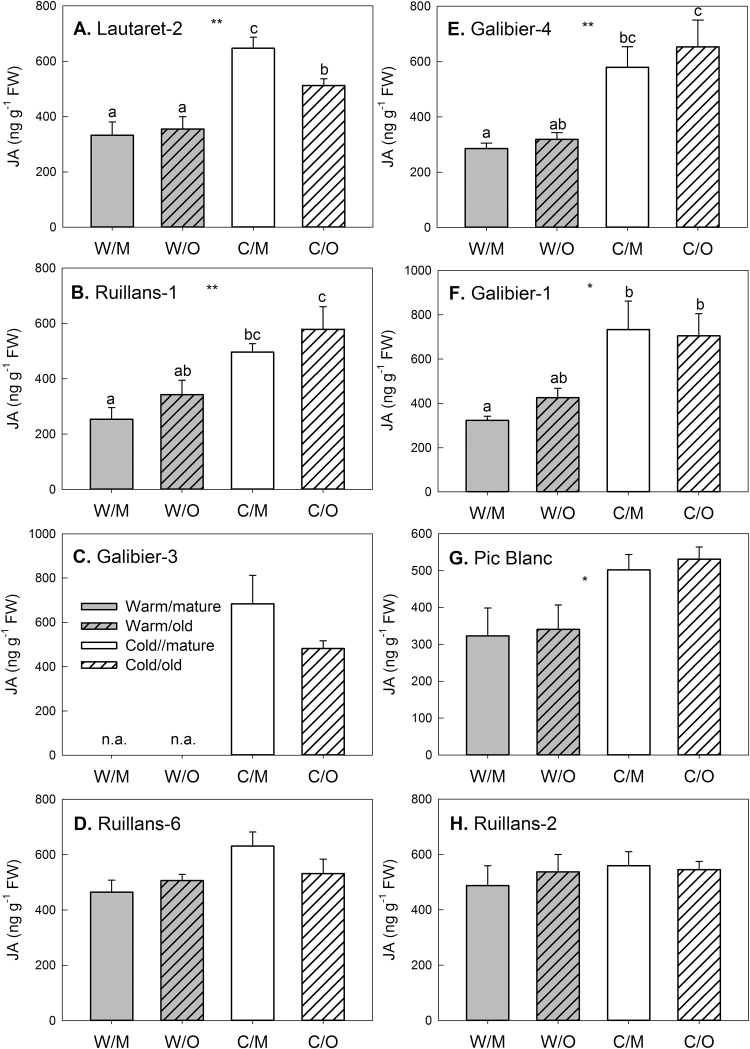
Jasmonic acid (JA) content on day 92 in the leaves of *A. alpina* plants grown continuously at 20 °C (warm, grey) or transferred to 5 °C on day 63 (cold, white). Locations are presented in order of increasing altitude of origin (A–H) Dependent on the accession (see Materials and methods) leaf 14 or leaf 16 was classified as mature (open bars) and leaf 12 or leaf 14 as old (hashed bars). W/M=warm/mature; W/O=warm/old.; C/M=cold/mature; C/O=cold/old. Data are means of leaves from three to five plants+SEM. Asterisks indicate statistically significant differences between the temperature treatments or age groups (ANOVA; * *P*≤0.05; ** *P*≤0.01); different letters indicate differences between the groups (Tukey’s test). n.a.=not available.

**Fig. 9.**
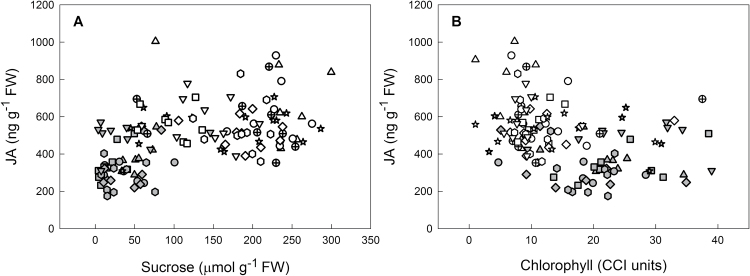
Relationship between jasmonic acid (JA) content on day 92 and sucrose (A) or chlorophyll (B) in the leaves of *A. alpina* plants grown continuously at 20 °C (warm, grey) or transferred to 5 °C on day 63 (cold, white). Symbols represent individual plants; different accessions are represented by different symbols. (A) Overall correlation: *r*=0.520, *P*≤ 0.001; warm: *r*=367, *P*=0.004; cold: *r*=–0.071, *P*=0.551. (B) Overall correlation: *r*=–0.383, *P*≤0.001; warm: *r*=–0.013, *P*=0.926; cold: *r*=–0.098, *P*=0.406.

Phytohormone contents were determined in the same samples as the sugar contents shown in Fig. 3. The contents of all phytohormones analysed (ABA, JA, SA, ACC, GA1, GA4, zeatin, ZR, 2iP, iPA, and IAA) are given in Supplementary Table S1. Although no major differences between the leaves of different age were found, chilling affected phytohormone contents. Content of the cytokinin zeatin (Fig. 6) was significantly higher in leaves of cold-grown than warm-grown plants of the accessions Galibier-1, Galibier-4, Lautaret-2, and Pic Blanc. Other cytokinins (ZR, 2iP, or iPA) were, however, not increased after cold treatment. IAA content (Fig. 7) was generally lower after cold treatment, and this effect was significant for the accessions Galiber-1, Galibier-4, Pic Blanc, and Ruillans-1. Overall (combining results for both temperatures), there was a highly significant negative correlation between IAA and zeatin contents (Supplementary Table S2). In the same accessions that contained less IAA after cold treatment, JA content was increased (Fig. 8). A negative correlation was found between IAA and JA, whereas there was a positive correlation between zeatin and JA for both temperature treatments combined (Supplementary Table S2). JA also correlated with sucrose content for both temperature treatments combined and when samples from warm-grown plants were treated separately, but not at cold temperature (Fig. 9A). Further, there was a statistically significant negative correlation between JA and chlorophyll contents, but only when both temperature treatments were combined (Fig. 9B). Other interesting relationships included a positive correlation between glucose and JA and between fructose and SA at warm temperature (Supplementary Table S2). Only few of the regression analyses between phytohormones and altitude of origin were statistically significant (Supplementary Table S3).

## Discussion

### Plants from higher altitudes have a larger capacity for accumulation of sucrose in response to chilling

Genotypes of *A. alpina* originating from higher altitudes accumulate more sucrose (Figs 3 and 4B; Supplementary Figs. S4 and S6B) and lose less chlorophyll in response to chilling, but senesce faster at warm temperature (Table 2). The differences in temperature response suggest that these adaptations to altitude are related to temperature, probably low day- or night-time temperatures during the growing season. Although daily maximum temperature at plant level was highly variable between sites, minimum temperature was related to altitude, decreasing by about 0.6–0.7°C per 100 m altitude change (Supplementary Fig. S1). The highest site is only snow-free from about July and can experience snowfall even in summer (Nivose La Meije, Meteo France, located about 20 m from the Ruillans-2 site), which further affects temperature and light availability.

Sugar content is correlated with freezing tolerance in different *Arabidopsis* accessions (Hannah *et al.*, 2006; Korn *et al.*, 2008; Zuther *et al.*, 2012). In addition to its osmoprotective function, sucrose was also shown to play a regulatory role during cold acclimation in *Arabidopsis* (Rekarte-Cowie *et al.*, 2008). In *Arabidopsis lyrata* ssp. *petraea* originating from different north European populations, sugars accumulated in response to cold treatment and differed between the populations, although this was not related to altitudinal or latitudinal gradients (Davey *et al.*, 2009). Within-species variation in sugar content dependent on altitude has also been documented for other species (e.g. Castrillo and Simoes, 1997; Molina-Montenegro *et al.*, 2012). However, in these studies samples were collected in the field and it is therefore not possible to draw any conclusions whether such variation was due to heritable adaptations or acclimation to the immediate growth environment. In *Calluna vulgaris* propagated from cuttings, sugar content correlated positively with altitude of origin (up to 250 m), but this was not found for *Erica cinerea* and *Erica tetralix* (Bannister, 1981). In *Espeleta schultzii*, sugar content increased with altitude, but only when expressed on a leaf area and not on a dry weight basis, which can be ascribed to smaller leaf area at higher altitude (Castrillo and Simoes, 1997). Here, the relationship between sucrose and altitude of origin at cold temperature held true when sugars were expressed on a fresh weight (Fig. 4B) and on a leaf area basis (Supplementary Fig. S6B), despite accessions from higher altitudes having smaller leaves (Supplementary Fig. S7). Importantly, plants were grown under controlled conditions after two rounds of propagation from seed, demonstrating a heritable basis of the variation and thus adaptation.

### Sucrose and temperature interact in the regulation of leaf senescence

At some time points after transfer to cold temperature chlorophyll content was positively correlated with altitude of origin (Table 2), which supports the idea that high-altitude plants may be better cold-adapted. However, sucrose may also have been responsible for the negative correlation between chlorophyll and altitude at warm temperature. The finding that sucrose treatment accelerated *A. alpina* senescence at warm, but not at cold temperature (Fig. 5) supports this conclusion. Induction of senescence by glucose was previously described (Wingler *et al.*, 2012*a*), but this effect was less pronounced than in *Arabidopsis*, where at warm temperature glucose, fructose, and sucrose all induce senescence to a similar extent. The lack of senescence induction by 3-*O*-methylglucose (Wingler *et al.*, 2012*b*) and delayed sugar-induced senescence in a hexokinase-1 mutant (Pourtau *et al.*, 2006) suggest that hexose-signalling is involved in senescence regulation in *Arabidopsis*. However, other sugar signalling pathways may also play a role, as shown by the requirement for trehalose-6-phosphate (Wingler *et al.*, 2012*b*), which serves as a signal for sucrose availability. Our results suggest that in *A. alpina* sucrose plays a more important role in senescence regulation than glucose.

Similar to the lack of effect of sucrose at cold temperature on senescence in *A. alpina*, cold temperature suppressed the senescence-inducing effect of glucose in *Arabidopsis* (Masclaux-Daubresse *et al.*, 2007). As overexpression of the C-repeat binding factor CBF2, a transcription factor involved in cold acclimation, delays developmental senescence, as well as induction of senescence by treatment with ABA, ethylene, salicylic acid, and methyl jasmonate (Sharabi-Schwager *et al.*, 2010), changes occurring during cold acclimation probably act downstream of sugar signalling and may involve phytohormone signalling pathways.

### Natural variation reveals synergistic and antagonistic relationships between phytohormones in response to chilling

Despite the role of phytohormones in senescence regulation, the effects of leaf age on phytohormone contents were less pronounced than effects of chilling (Figs 6–8; Supplementary Table S1). In addition, no significant correlations between chlorophyll and phytohormone contents were found, when each temperature was treated separately (Supplementary Table S2). However, when both temperatures were combined, there was e.g. a negative correlation between JA and chlorophyll content, in agreement with higher JA and lower chlorophyll in the cold-treated plants. A sustained increase in JA in response to chilling was also reported for winter wheat (Kosová *et al.*, 2012), and in *Arabidopsis* jasmonate was shown to enhance freezing tolerance by inducing the ICE-CBF/DREB1 cold response pathway (Hu *et al.*, 2013). Our findings support a role of JA in cold acclimation in *A. alpina*. However, no correlation between JA content and altitude of origin was found (Supplementary Table S3), demonstrating that adaptation to higher altitude does not involve an enhanced capacity to accumulate JA during chilling.

In addition to changes in JA, zeatin content increased with cold treatment, whereas IAA content decreased. The role of cytokinins and auxins in cold acclimation has not entirely been resolved. In wheat, changes in the contents of these phytohormones were dependent on genotype and time after transfer to cold temperature (Kosová *et al.*, 2012). At warm temperature, zeatin was negatively correlated with IAA content. This can probably be explained with interactions between the production and transport of cytokinin and auxins, which affects organ dominance (Bangerth *et al.*, 2000). The negative correlation may therefore reflect architectural differences between the accessions in response to chilling.

In addition to the antagonism between zeatin and IAA, our results show a positive relationship between zeatin and JA and a negative one between auxin and JA. Although the signalling function of auxin and jasmonates may be antagonistic rather than synergistic, treatment with JA can induce cytokinin accumulation and vice versa (O’Brien and Benková, 2013). Our results support the idea that there is synergism in the biosynthetic pathways *in planta*. In contrast to the opposite effect on auxin and JA found here, both phytohormones were increased in response to cold stress in rice (Du *et al.*, 2013). The negative correlation in *A. alpina* at warm temperature suggests a role during senescence. In agreement with this, auxin and JA have recently been shown to have antagonistic effects on senescence, with auxin acting as a repressor of JA-induced senescence via the senescence-repressing transcription factor WRKY57 (Jiang *et al.*, 2014).

Despite the well-established role of ABA during senescence and stress, ABA content was only affected in Galibier-1 and Ruillans-1, where it was increased in old leaves in response to chilling (Supplementary Table S1). Cold acclimation can occur via ABA-dependent and ABA-independent pathways. It was shown that ABA only increases transiently within the first days of cold treatment and then declines (Kosová *et al.*, 2012), suggesting that it is involved in the initial cold response process but not required to maintain the cold-acclimated state.

### Natural variation reveals interactions between sugar and phytohormone contents

Given the crosstalk between sugar and phytohormone signalling pathways (Gibson, 2004) and the role of phytohormones in senescence regulation, interactions were expected between sugar and phytohormone accumulation. Although cytokinins generally delay senescence, they do not seem to inhibit sugar-induced senescence (Wingler *et al.*, 1998). The lack of a senescence-inducing effect of sucrose at cold temperature (Fig. 5) can therefore probably not be ascribed to the zeatin accumulation during chilling (Fig. 6).

JA content was positively correlated with sucrose and total sugar contents at warm, but not at cold temperature and overall negatively correlated with chlorophyll content (Fig. 9). In *Arabidopsis* seedlings JA accumulation was shown to be sugar dependent (Hamann *et al.* 2009): osmotic stress by sorbitol treatment increases JA accumulation in the presence of sucrose. A requirement for sugars for JA synthesis is also in agreement with changes in the expression of JA biosynthesis genes during senescence. In maize leaves, senescence can be induced by preventing pollination, which results in sugar accumulation and enhanced expression of JA biosynthesis genes (Sekhon *et al.*, 2012). In *Arabidopsis*, the jasmonate biosynthetic pathway is up-regulated during natural senescence, but not during dark-induced senescence (van der Graaff *et al*., 2006). However, Seltmann *et al.* (2010) reported strong accumulation of JA in dark-induced senescence of detached leaves, which should result in sugar deprivation (but may also involve a wounding-dependent response), and jasmonate signalling seems to be involved in dark-induced senescence (Buchanan-Wollaston *et al.*, 2005). Overall, the exact role of jasmonates in senescence regulation remains unclear, and it has been demonstrated that their signalling function is dependent on age-related changes (Jibran *et al.*, 2013).

Jasmonates and sucrose also interact in the regulation of the synthesis of anthocyanins, which typically accumulate during senescence and cold acclimation. Synergistic effects between sucrose and jasmonates were reported for *Arabidopsis* (Loreti *et al.*, 2008): whereas JA on its own does not induce anthocyanin accumulation, it enhances sucrose-dependent anthocyanin biosynthesis in seedlings, probably by synergistic regulation of expression of the transcription factors genes *PAP1* and *PAP2*. Our results suggest interactions between sucrose and jasmonate biosynthesis. However, as there was no negative correlation between JA and chlorophyll content at warm temperature (Supplementary Table S2), synergistic effects between sucrose and jasmonates are unlikely to play a role in senescence regulation, despite clear interactions between the extent of senescence and sucrose accumulation (Fig. 4 and Supplementary Fig S6).

Abscisic acid (ABA) is also known to induce senescence, and a substantial overlap between glucose- and ABA-regulated gene expression was found in *Arabidopsis* (Li *et al.*, 2006). At warm temperature, there was a positive correlation between glucose and ABA contents, which is not surprising as glucose treatment results in the accumulation of ABA (Arenas-Huertero *et al.*, 2000). In addition, ABA synthesis and signalling are required for glucose sensitivity of seedlings (Rook and Bevan, 2003). Although these signalling interactions have mainly been characterised in *Arabidopsis* seedlings, more recent work also demonstrates a role in mature leaves: under conditions of high sugar availability hexokinase, which acts as a sugar sensor and predominantly phosphorylates glucose, stimulates stomatal closure in an ABA-dependent manner (Kelly *et al.*, 2013). However, ABA does not seem to be involved in glucose signalling during senescence (Pourtau *et al.*, 2004). It is therefore possible that the glucose/ABA interactions demonstrated here are involved in stomatal control.

In conclusion, our work demonstrates that physiological adaptations, including enhanced capacity to accumulate sucrose, occur over an altitudinal gradient in the alpine perennial *A. alpina*. It is also shown that sucrose accumulation is likely to play different roles, dependent on the temperature—inhibiting stress-induced senescence at cold temperature, but accelerating developmental senescence at warm temperature. In addition, analysing natural variation has enabled us to identify relationships between phytohormone and sugar contents. The natural variation in these physiological parameters can be used to explore the genetic basis of cold response and senescence regulation.

## Supplementary data

Supplementary data are available at *JXB* online

Table S1. Phytohormone contents in leaves of 92-day-old plants.

Table S2. Correlations between phytohormone, sugar and chlorophyll contents.

Table S3. Regression analysis of the relationship between phytohormone contents and altitude of origin.

Figure S1. Daily minimum and maximum temperatures during the growing season.

Figure S2. Maximum photosystem II efficiency (F_v_/F_m_) in the leaves of *A. alpina* accessions grown continuously at 20 °C (warm) or transferred to 5 °C on day 63 (cold).

Figure S3. Comparison of chlorophyll content between accessions of *A. alpina* grown continuously at 20 °C (warm) or transferred to 5 °C on day 63 (cold).

Figure S4. Glucose, fructose and sucrose contents on day 94 in the leaves of *A. alpina* plants grown continuously at 20 °C or transferred to 5 °C on day 63.

Figure S5. Chlorophyll content on day 93 in the leaves of *A. alpina* plants grown continuously at 20 °C or transferred to 5 °C on day 63.

Figure S6. Relationship between sucrose content on day 94 and chlorophyll (A) or altitude of origin (B) in the leaves of *A. alpina* plants grown continuously at 20 °C or transferred to 5 °C on day 63.

Figure S7. Leaf size in relation to altitude of origin.

Supplementary Data
