# Supplementary material for: Adaptation to altitude affects the senescence response to chilling in the perennial plant Arabis alpina
Source: J Exp Bot. 2014 Nov 4;66(1):355–67. doi: 10.1093/jxb/eru426 (PMC4265169; doi:10.1093/jxb/eru426)
Supplement: Supplementary Data [file supp_66_1_355__index.html]

Adaptation to altitude affects the senescence response to chilling in the perennial plant Arabis alpina — Adaptation to altitude affects the senescence response to chilling in the perennial plant Arabis alpina — Supplementary Data 

# Adaptation to altitude affects the senescence response to chilling in the perennial plant *Arabis alpina*

## Supplementary Data

Data files

**Files in this Data Supplement:**

- Supplementary Data - Supplementary Data
- Supplementary Data - Supplementary Data
- Supplementary Data - Supplementary Data
