# Supplementary material for: Adaptation to altitude affects the senescence response to chilling in the perennial plant Arabis alpina
Source: J Exp Bot. 2014 Nov 4;66(1):355–67. doi: 10.1093/jxb/eru426 (PMC4265169; doi:10.1093/jxb/eru426)
Supplement: Supplementary Data [file supp_eru426_jexbot133728_file001.pdf]

Adaptation to altitude affects the senescence response to chilling in the perennial plant *Arabis alpina*

Astrid Wingler[1,*], Marta Juvany[2], Caroline Cuthbert[1] and Sergi Munné-Bosch[2]

[1]Genetics, Evolution and Environment, University College London, Gower Street, London WC1E 6BT, United Kingdom.

[2]Departament de Biologia Vegetal, Facultat de Biologia, Universitat de Barcelona, Avinguda Diagonal 643, E-08028 Barcelona, Spain.

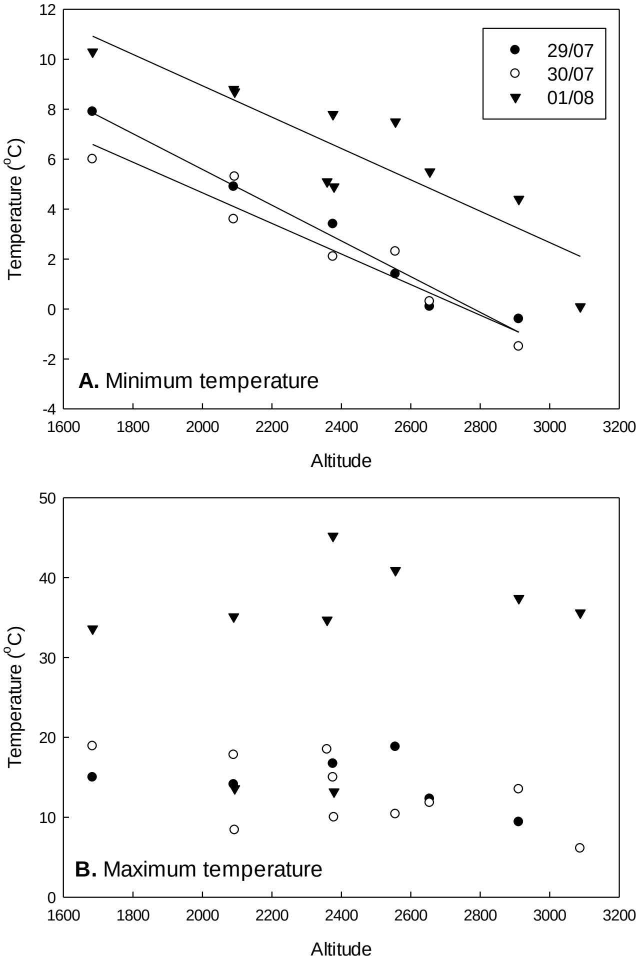

Supplementary Fig. S1. Daily minimum (**A**) and maximum (**B**) temperatures determined at plant level on three different dates during the growing season in 2014. Linear regressions were found between minimum temperatures and altitude. The sites are, with increasing altitude: Romanche (1684 m), Lautaret-2 (2090 m), Lautaret-1 (2093 m), Ruillans-1 (2359 m), Galibier-3 (2376 m), Ruillans-6 (2379 m), Galibier-1 (2556 m), Galibier-4 (2655 m), Pic Blanc (2911 m), Ruillans-2 (3088 m). 29/07: R^2 = 0.913, p < 0.001, -0.613°C/100 m. 30/07: R^2 = 0.976, p < 0.001, -0.716°C/100 m. 01/08: R^2 = 0.775, p < 0.001, -0.628°C/100 m.

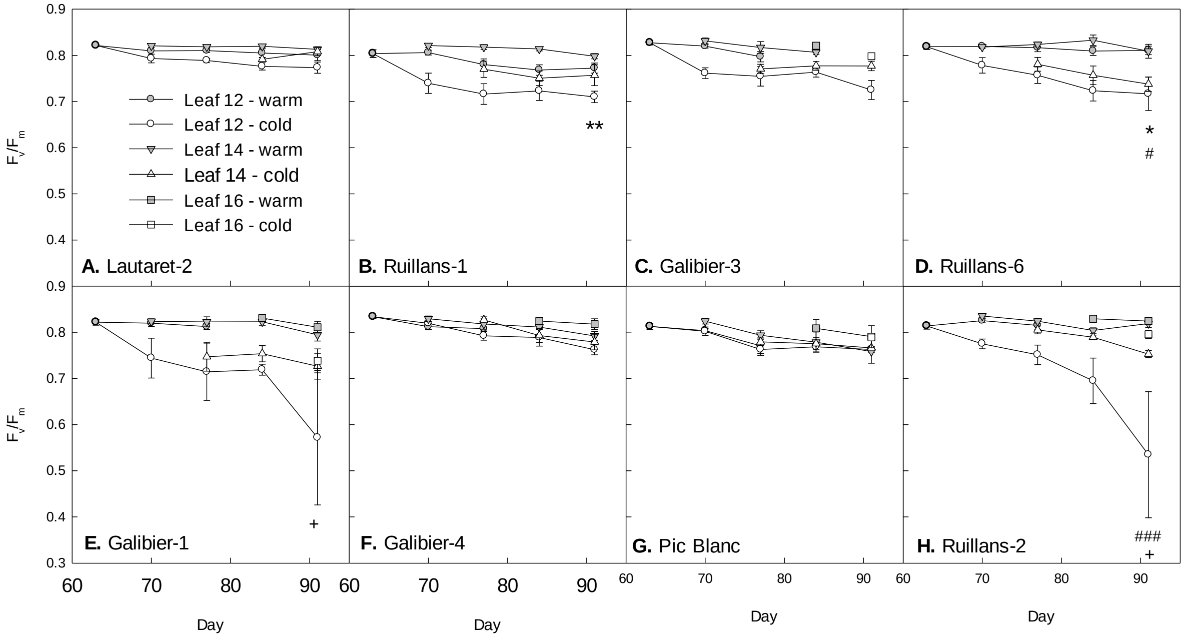

Supplementary Fig. S2. Maximum photosystem II efficiency (F_v/F_m) in the leaves of *A. alpina* accessions grown continuously at 20°C (warm) or transferred to 5°C on day 63 (cold). L12-W = leaf 12, warm (grey circles); L12-C = leaf 12, cold (white circles); L14-W = leaf 14, warm (grey triangles); L14-C = leaf 14 cold (white triangles); L16-W = leaf 16, warm (grey squares); L16-C = leaf 16 cold (white squares). Data are means of leaves from three to five plants ± SE. t-tests were performed to compare data from warm- and cold-treated plants at the final timepoint; asterisks indicate statistically significant differences between leaves 12 (* $p \leq 0.05$; ** $p \leq 0.01$; *** $p \leq 0.001$); hash signs indicate statistically significant differences between leaves 14 ($^{\#}p \leq 0.05$; $^{\#\#}p \leq 0.01$; $^{\#\#\#}p \leq 0.001$); plus signs indicate statistically significant differences between leaves 16 ($^{+}p \leq 0.05$; $^{++}p \leq 0.01$; $^{+++}p \leq 0.001$).

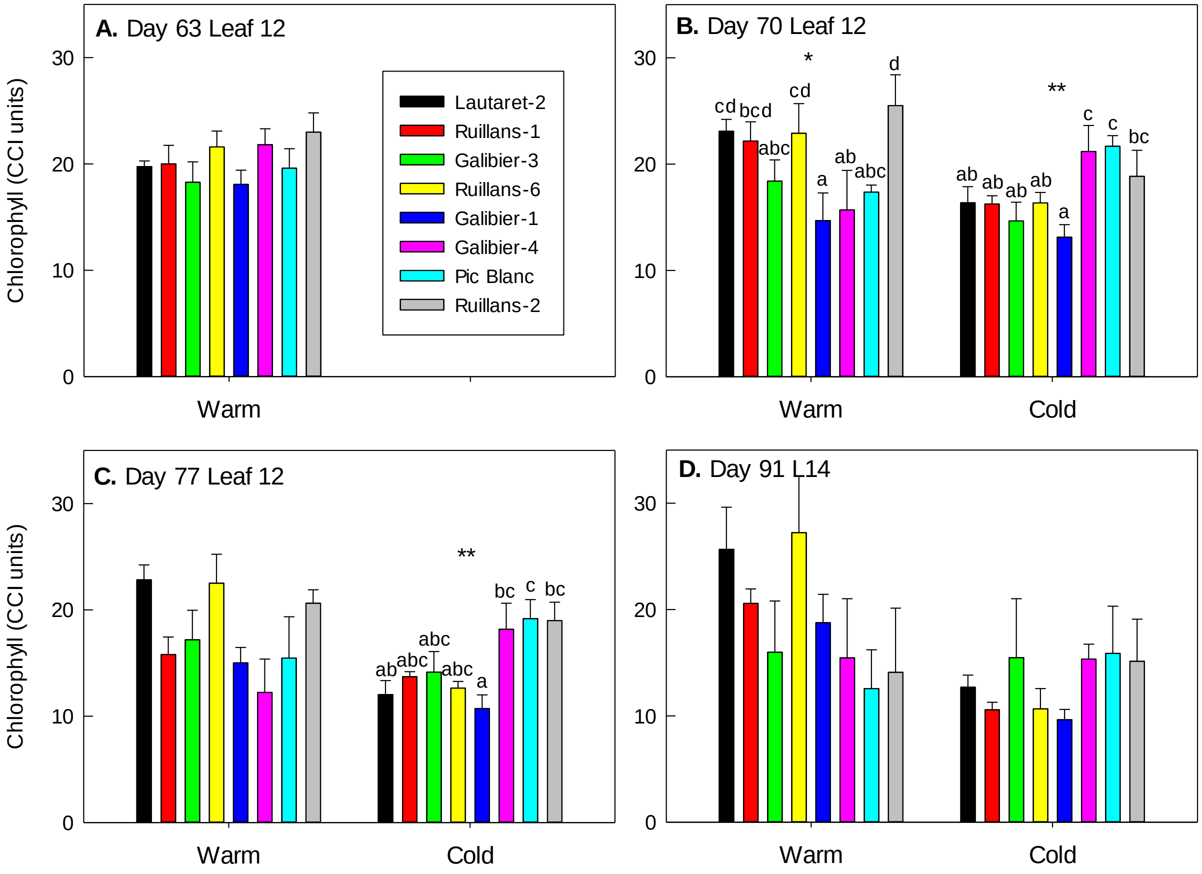

Supplementary Fig. S3. Comparison of chlorophyll content between accessions of *A. alpina* grown continuously at 20°C (warm) or transferred to 5°C on day 63 (cold). Data are means of leaves from three to five plants + SE. Asterisks indicate statistically significant differences between the accessions (ANOVA; * $p \leq 0.05$; ** $p \leq 0.01$); different letters indicate differences between the groups (Tukey test).

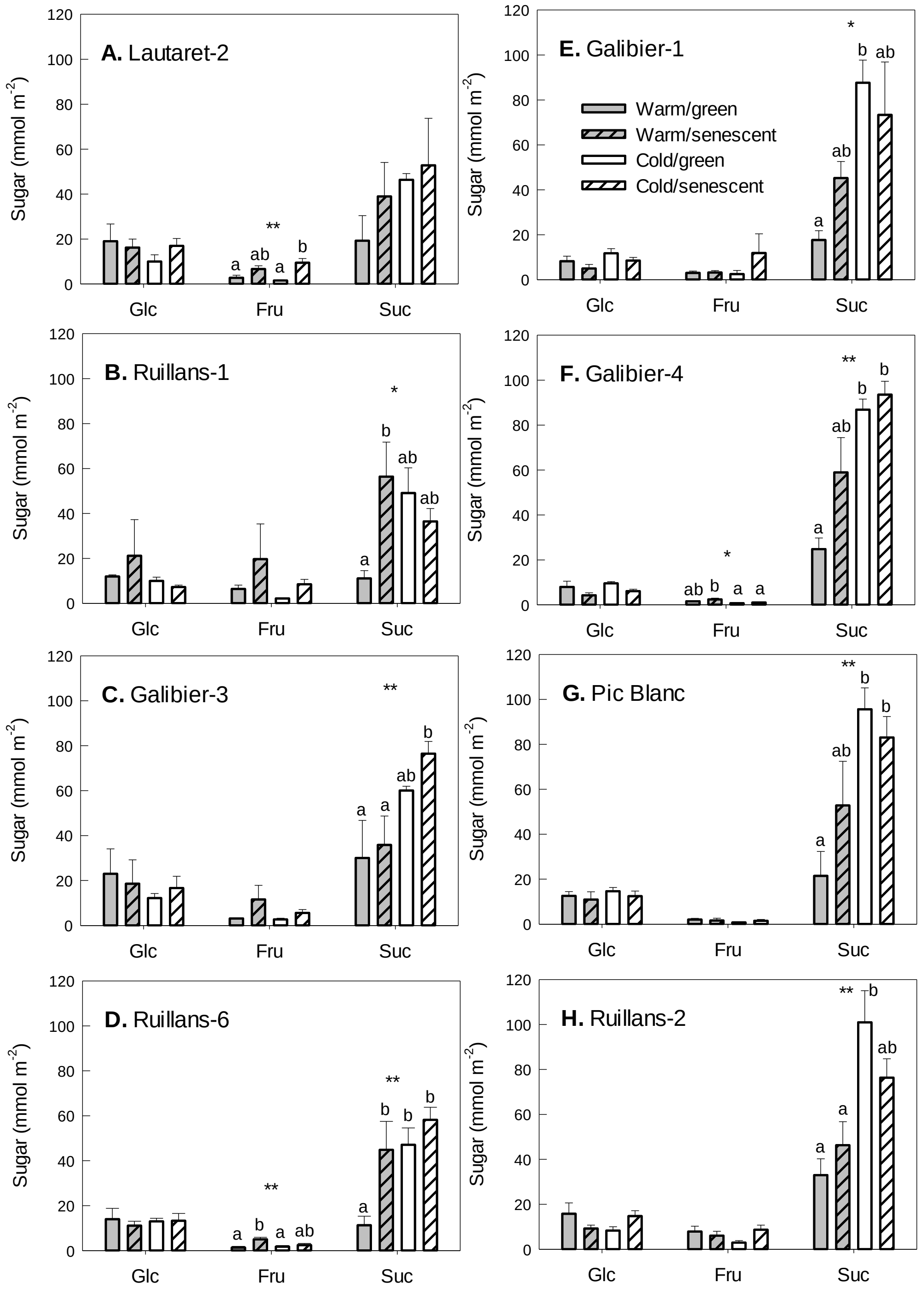

Supplementary Fig. S4. Glucose, fructose and sucrose contents on day 94 in the leaves of *A. alpina* plants grown continuously at 20°C (warm, grey) or transferred to 5°C on day 63 (cold, white). Leaves were classified into green (open bars) or senescent (hashed bars) based on chlorophyll content (see Supplementary Fig. S4). Data are means of leaves from two to five plants + SE. Asterisks indicate statistically significant differences between the temperature treatments or age groups (ANOVA; * $p \leq 0.05$; ** $p \leq 0.01$); different letters indicate differences between the groups (Tukey test). n.a. = not available.

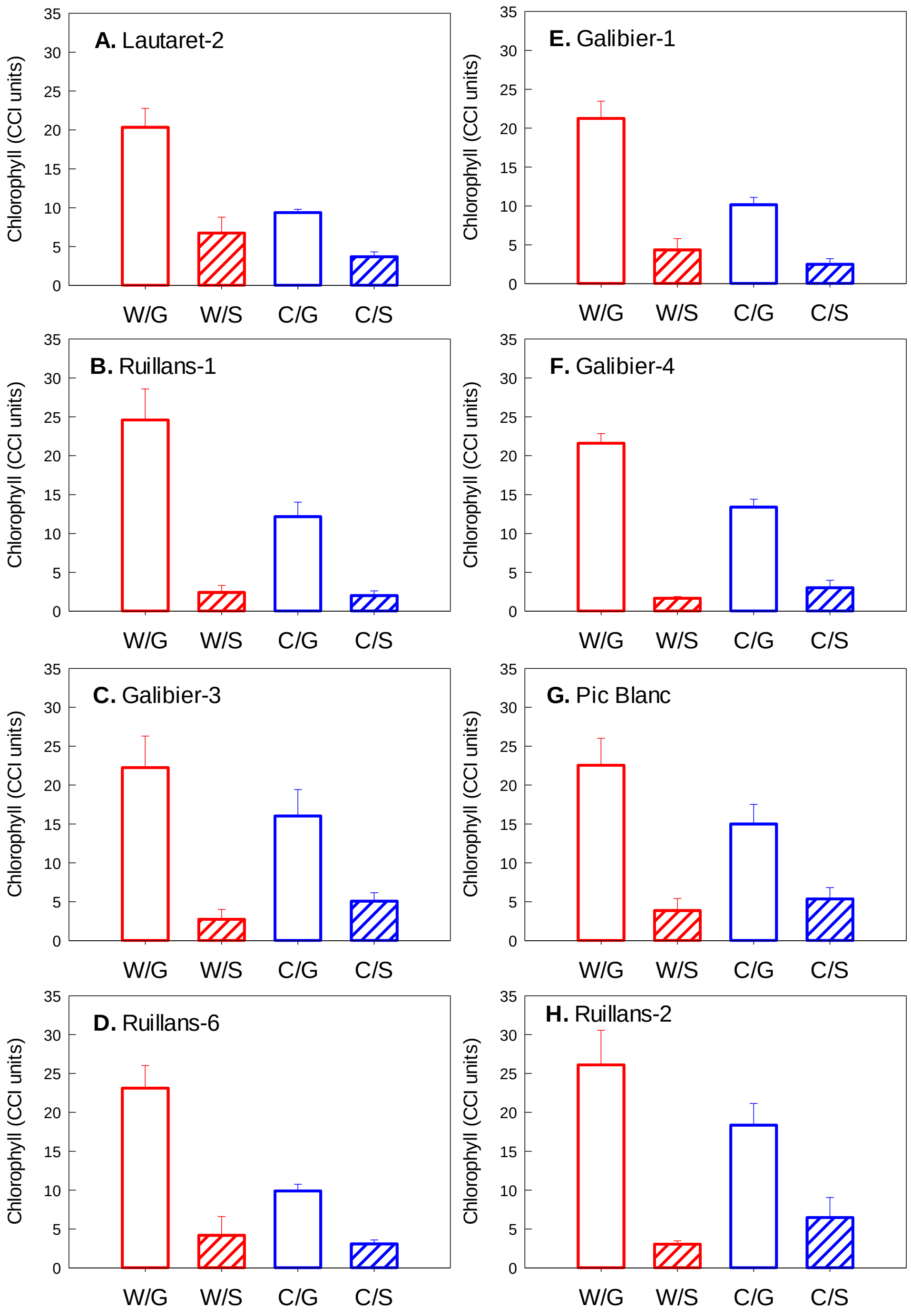

Supplementary Fig. S5. Chlorophyll content on day 93 in the leaves of *A. alpina* plants grown continuously at 20°C (warm, red) or transferred to 5°C on day 63 (cold, blue). Leaves were classified into green (open bars) or senescent (hashed bars). Data are means of leaves from two to five plants + SE.

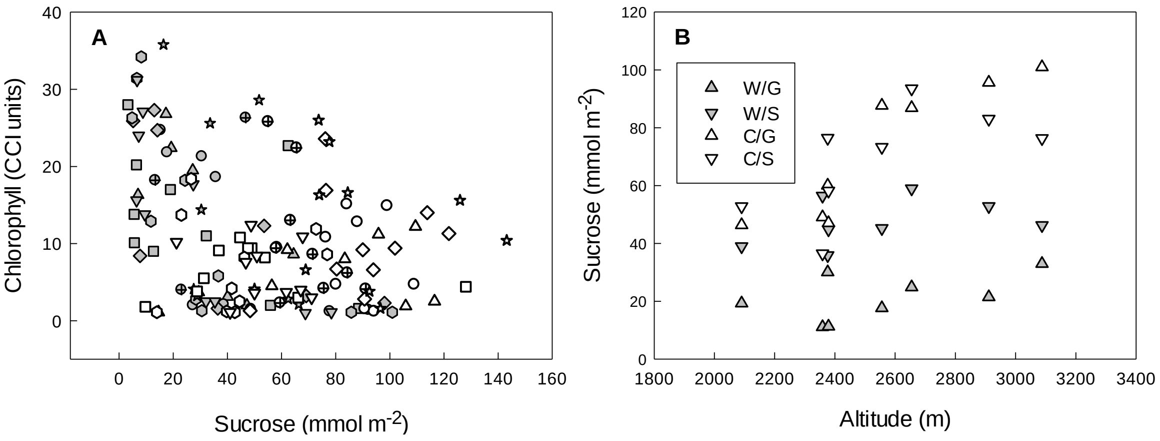

Supplementary Fig. S6. Relationship between sucrose content on day 94 and chlorophyll (**A**) or altitude of origin (**B**) in the leaves of *A. alpina* plants grown continuously at 20°C (warm, grey) or transferred to 5°C on day 63 (cold, white). **A.** Symbols represent individual plants; different accessions are represented by different symbols. Overall correlation: $r = -0.368$, $p \leq 0.001$; warm: $r = -0.602$, $p \leq 0.001$; cold: $r = 0.136$, $p = 0.241$. **B.** Replicate plants of each accession and leaf developmental stage were averaged for regression analysis. Regression warm: $R^2 = 0.052$, $p = 0.393$; cold: $R^2 = 0.599$, $p \leq 0.001$. W/G = warm/green; W/S = warm/senescent; C/G = cold/green; C/S = cold/senescent.

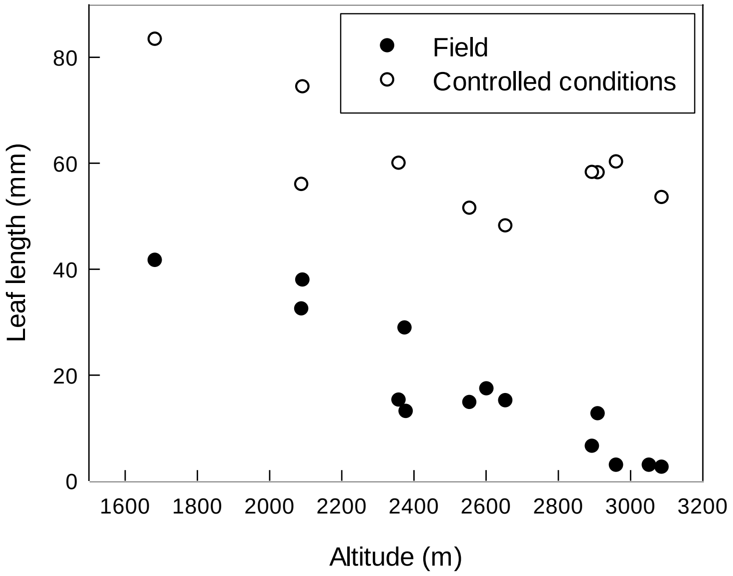

Supplementary Fig. S7. Length of the largest leaf of *A. alpina* plants originating from different altitudes. Measurements were done in the field (closed circles) and after growth from seed under controlled (warm) conditions (open circles). Each point is the mean of at least three plants from each location. Regression field: R^2 = 0.866, p < 0.000; controlled conditions: R^2 = 0.4654, p = 0.015.
